# Comparison of Choroidal Thickness, Foveal Avascular Zone, and Macular Capillary Density in Macular Edema Secondary to Branch Retinal Vein Occlusion Treated with Ranibizumab or Aflibercept—A Prospective Study

**DOI:** 10.3390/medicina58040540

**Published:** 2022-04-14

**Authors:** Yu-Te Huang, I Wang, Chun-Ju Lin, Chun-Ting Lai, Ning-Yi Hsia, Huan-Sheng Chen, Peng-Tai Tien, Henry Bair, Jane-Ming Lin, Wen-Lu Chen, Chang-He Chen, Wen-Chuan Wu, Yi-Yu Tsai

**Affiliations:** 1 Department of Ophthalmology, China Medical University Hospital, China Medical University, Taichung 404, Taiwan; tonyhuang791112@gmail.com (Y.-T.H.); u9801310@cmu.edu.tw (I.W.); deepwhite1111@hotmail.com (N.-Y.H.); u702054@hotmail.com (P.-T.T.); d4301@seed.net.tw (J.-M.L.); joejayjoejay2000@yahoo.com.tw (W.-L.C.); dannychen28@gmail.com (C.-H.C.); wuoph@kmu.edu.tw (W.-C.W.); yiyutsai@seed.net.tw (Y.-Y.T.); 2 School of Medicine, College of Medicine, China Medical University, Taichung 404, Taiwan; 3 Department of Optometry, Asia University, Taichung 413, Taiwan; 4 An-Shin Dialysis Center, NephroCare Ltd., Fresenius Medical Care, Taichung 401, Taiwan; dalusoha@gmail.com; 5 Graduate Institute of Clinical Medical Science, College of Medicine, China Medical University, Taichung 404, Taiwan; 6 Byers Eye Institute, Stanford University School of Medicine, Stanford, CA 94303, USA; hbair@stanford.edu

**Keywords:** branch retinal vein occlusion, enhanced depth imaging optical coherence tomography, foveal avascular zone, macular capillary density, macular edema, optical coherence tomography angiography, aflibercept, ranibizumab

## Abstract

This prospective comparative case series aims to compare best-corrected visual acuity (BCVA), retinal microvasculature, and retinal structural changes in patients treated with either ranibizumab or aflibercept for macular edema (ME) secondary to treatment-naïve branch retinal vein occlusion (BRVO) by optical coherence tomography angiography (OCTA). Ten patients were enrolled with macular capillary density of the superficial capillary plexus (SCP) and deep capillary plexus (DCP) and foveal avascular zone (FAZ) measured in both eyes before and after treatment. Final central retinal thickness and BCVA improved significantly (*p* < 0.05), and densities of SCP and DCP of BRVO sectors were significantly lower at baseline than fellow eye counterparts and remained persistently lower during treatment, particularly in the aflibercept group (*p* < 0.05). SCP density, DCP density of both BRVO sectors (*p* = 0.0001, *p* < 0.0001), and non-BRVO sectors (*p* < 0.0001, *p* < 0.0001) were significantly correlated with final BCVA for diseased eyes. Using multivariate general linear model analysis, and including OCTA parameters only, but not all of the available clinical data, DCP density of BRVO sectors in both eyes was the most predictive factor for final visual outcome (probability *p* < 0.0001). OCTA offered further qualitative and quantitative evaluation of treatment-naïve BRVO. Judging by OCTA parameters, not only in the diseased eye but also in the fellow eye, DCP density of BRVO sectors was the most predictive factor of final visual outcome.

## 1. Introduction

Retinal vein occlusion is second in prevalence only to diabetic retinopathy among retinal vascular disorders and is a major cause of vision loss worldwide [1,2,3,4]. Branch retinal vein occlusion (BRVO) can lead to numerous complications, with macular edema (ME) being the most common and most important cause of vision loss [5,6]. Although 18–41% of BRVO-induced ME (BRVO-ME) resolve spontaneously over time [2], the extended period of hypoxia resulting from ME can lead to irreversible loss of vision even in such cases.

The mainstay of treatment for BRVO is pharmacotherapy with anti-vascular endothelial growth factors (anti-VEGF) [7] including ranibizumab (Lucentis, Genentech Inc., South San Francisco, CA, USA) [8] and aflibercept (EYLEA-Regeneron Pharmaceuticals, Inc., Tarrytown, New York, NY, USA, and Bayer Healthcare Pharmaceuticals, Berlin, Germany) [9], or corticosteroids such as a dexamethasone intravitreal implant (Ozurdex, Allergan, Irvine, CA, USA) [10].

Traditionally, BRVO can be evaluated by fundus photography, enhanced depth imaging-optical coherence tomography (EDI-OCT), and fluorescein angiography (FA) [11]. Optical coherence tomography angiography (OCTA) uses the split-spectrum amplitude–decorrelation angiography algorithm to detect erythrocyte movement and can image the capillary network in a noninvasive manner [12,13,14,15]. OCTA provides images of the bloodstream in various layers of the retina, providing valuable data for BRVO patients, especially in the deep capillary plexus (DCP). Previous studies have demonstrated DCP to be the critical area of vascular changes, and decreased perfusion in this area significantly affects visual prognosis [16,17].

In this prospective study, we aimed to compare changes in OCTA parameters of patients treated with either ranibizumab or aflibercept for treatment-naïve BRVO-ME. In addition, we sought to identify possible predictive factors of visual outcome after treatment.

## 2. Materials and Methods

### 2.1. Study Population

This was a prospective comparative case series. From 1 January 2018 to 1 January 2020, consecutive treatment-naïve patients with BRVO-ME who were candidates for intravitreal anti-VEGF therapy were collected. Inclusion criteria were patients over 20 years old with follow-up periods longer than 12 months and with BRVO demonstrated by FA, with ME greater than 300 μm. Symptom onset must occur within three months without any previous treatments. An OCTA image quality of at least seven during follow-up was required to be included. Exclusion criteria were as follows: follow-up periods less than 12 months or a history of poorly controlled hypertension (random systolic blood pressure over 200 mmHg), diabetes mellitus (HbA1c over 10.0%), stroke, transient ischemic attack, recent cardiac arrest, or pregnancy or breast feeding at the time of disease [18,19] Patients with uveitis, uncontrolled glaucoma, vitreous hemorrhage, vitreomacular traction, any evidence of fibrovascular proliferation in the macular area, or significant media opacity affecting the quality of the images. Patients with a history of intraocular infection or allergy to the drugs were also excluded.

### 2.2. Study Protocol

Patients enrolled in this study were randomized to receive either the ranibizumab or aflibercept treatment. The treatment regimen was monthly loading doses for three months followed by pro re nata injections. The patients were followed up monthly with comprehensive ophthalmologic examination including BCVA, intraocular pressure (IOP), SD-OCT, EDI-OCT, and OCTA, in addition to blood pressure measurements. Possible complications were monitored.

### 2.3. Assessment of Clinical Outcome

Central retinal thickness (CRT) and central choroidal thickness (CCT) were measured by SD-OCT and EDI-OCT (Spectralis, Heidelberg, Germany). For the detailed measurement of CCT, we used the similar methods previously described [20]. The lower segmentation line that originally corresponded to the lower border of the RPE-Bruch’s membrane complex was moved down to the choroid-scleral boundary. And the upper segmentation line that originally corresponded to the ILM was moved down to the lower border of the RPE-Bruch’s membrane complex. The software subsequently calculated the mean choroidal thickness. Manual adjustment of the segmentation lines on each OCT B-scan was independently carried out by trained and experienced technicians and confirmed by the senior investigators using a proprietary OCT viewing software (Heidelberg Eye Explorer version 1.7.1.0). OCTA was performed using an RTVue XR 100 Avanti instrument (Optovue, Inc., Fremont, CA, USA). For each eye, a 3 × 3-mm scan centering on the fovea was acquired. Automated OCT segmentation was performed using the Angio-Vue module. All images were taken at baseline (one to seven days before injections) and every four weeks. In addition, high-quality images (quality score beyond seven) were required during the follow up.

Foveal avascular zone (FAZ), non-perfusion area (NPA), and density of macular capillaries of the superficial capillary plexus (SCP) and DCP were automatically generated by the instruments. The SCP enface image was segmented with an inner boundary at three μm beneath the internal limiting membrane and an outer boundary set at 15 μm beneath the inner plexiform layer, whereas the DCP enface image was segmented with an inner boundary 15 μm beneath the inner plexiform layer and an outer boundary at 70 μm beneath the inner plexiform layer. Vessel density was calculated as the proportion of the measured area occupied by blood vessels with flow, defined as pixels having correlation values above the threshold level. The fovea was defined as the area within the central 1-mm ring of the Early Treatment Diabetic Retinopathy Study (ETDRS) grid.

### 2.4. Statistical Analysis

A statistical analysis was performed using SPSS software (version 16; SPSS, Inc., Chicago, IL, USA). A paired *t*-test was used for quantitative data analysis before and after injections. A Mann-Whitney U test was used for comparison of the mean changes between the two groups (Aflibercept and Ranibizumab). *p*-values less than 0.05 were considered significant. A Pearson correlation analysis was performed to detect possible correlation between OCTA parameters and BCVA. A multivariate general linear model (GLM) analysis was used to confirm the predictive parameters influencing BCVA outcome.

## 3. Results

Eighteen patients were evaluated at the initiation of the study. However, three patients did not meet the inclusion criteria, as one patient had CRVO, another had a history of glaucoma, and one had symptoms for more than three months. One patient failed to return to follow up due to personal reasons. Follow-up for four patients violated protocol, as OCTA was not performed during every outpatient follow-up.

Ten eyes of 10 patients (mean age 61.4 years; males 50%) were eventually included. Half of the eyes were treated with ranibizumab and the other half with aflibercept. The baseline data did not significantly differ between the ranibizumab and aflibercept groups (Table 1).

### 3.1. CRT Changes

**Figure 1:**
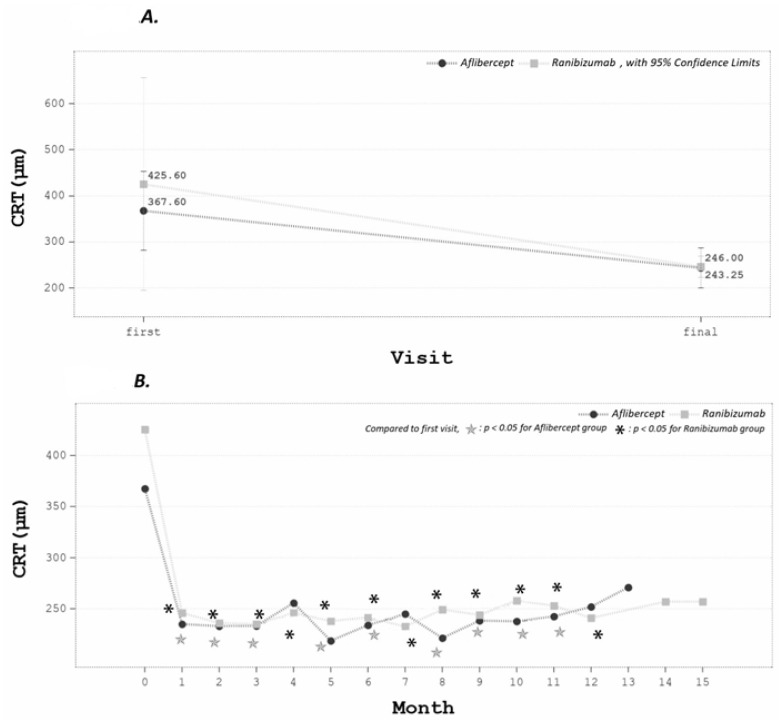
(**A**) The CRT improved significantly. (**B**) From serial follow-up data, CRT significantly improved since the first follow-up month.

After anti-VEGF treatment, mean final CRT decreased significantly from 396.60 ± 135.64 to 244.63 ± 20.29 μm (*p* < 0.05). From serial follow-up data, CRT significantly decreased beginning from the first month of follow-up in both groups (Figure 1).

### 3.2. BCVA Changes

**Figure 2:**
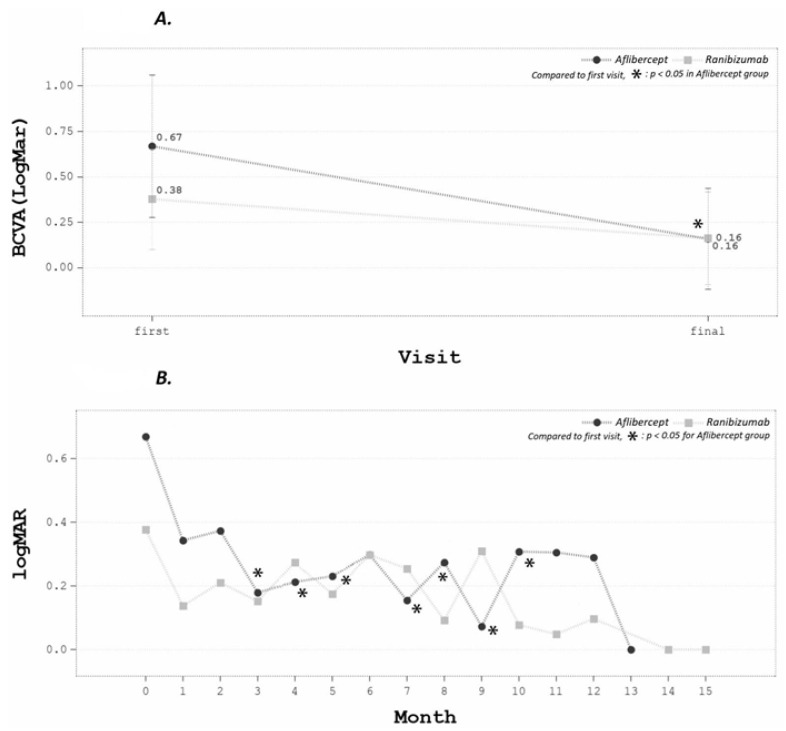
(**A**) The BCVA improvement was more prominent in the aflibercept group. (**B**) From serial follow-up data, BCVA significantly improved since the first follow-up month. In subgroup analysis, the improvement mainly occurred in the aflibercept group.

Overall, the mean final LogMAR BCVA improved significantly from 0.52 ± 0.30 to 0.16 ± 0.20 (*p* < 0.05). The improvement was more prominent in the aflibercept group (Figure 2A). From serial follow-up data, BCVA significantly improved beginning from the first month of follow-up, although a subgroup analysis found that the improvement mainly occurred in the aflibercept group (Figure 2B).

### 3.3. SCP and DCP Changes

**Figure 3:**
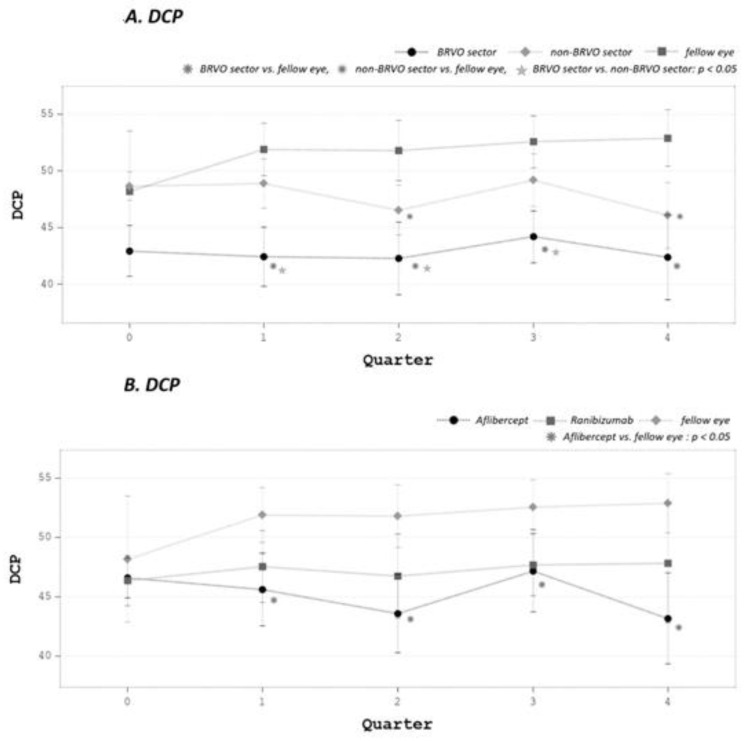
(**A**) DCP in BRVO sectors remained low after the initiation of treatment and reached significance compared with non-BRVO sectors and fellow eye (*p* < 0.05). (**B**) In subgroup analysis, the significance was mostly in the aflibercept group.

SCP and DCP changes before and after treatment are summarized in Table 2. Densities of the SCP and DCP in the BRVO sectors were lower compared with that of the fellow eye (*p* = 0.035 in both group). DCP density in BRVO sectors remained low after the initiation of treatment, compared with non-BRVO sectors and the fellow eye (*p* < 0.05) (Figure 3A). With subgroup analysis, statistical significance was only present in the aflibercept group (Figure 3B).

**Figure 4:**
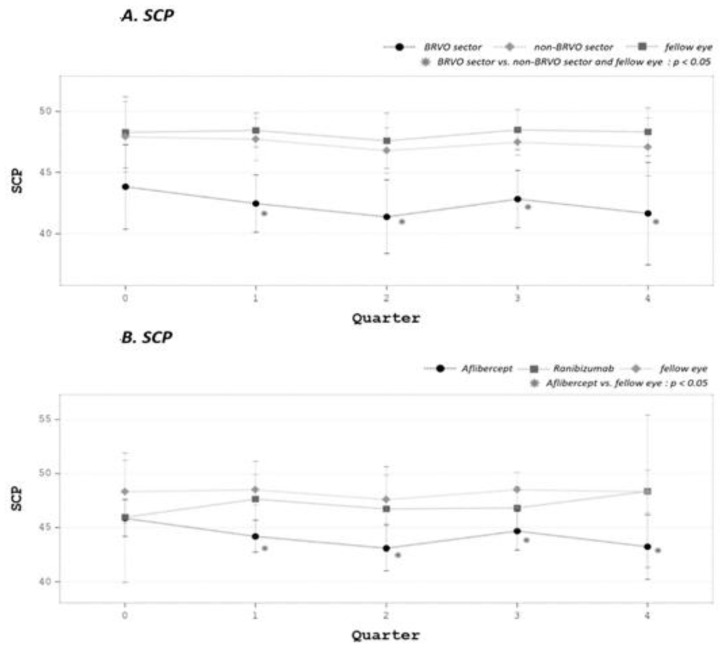
(**A**) SCP in BRVO sectors remained low after the initiation of treatment and reached significance compared with non-BRVO sectors and fellow eye (*p* < 0.05). (**B**) In subgroup analysis, the significance was mostly in the aflibercept group.

After treatment, SCP densities decreased in both eyes and showed no significant differences in BRVO sectors compared with the fellow eye (*p* = 0.12), whereas DCP remained significantly affected compared with the fellow eye (*p* = 0.002) (Figure 4A,B). Neither of the treatments increased SCP or DCP density in the diseased eye (both in BRVO sectors and as a whole).

### 3.4. FAZ, NPA, ORFA Changes

In diseased eyes, the final NPA significantly increased from 19.16 ± 4.74 to 23.97 ± 2.60 mm^2^ (*p* < 0.05) after anti-VEGF treatment (Table 2). FAZ and the outer retina flow area (ORFA) of the diseased eyes did not significantly differ from the fellow eye during the follow-up period in either the aflibercept or ranibizumab groups.

**Table 2:** NPA, FAZ, SCP, DCP changes in Diseased Eye and Fellow Eye.

	Diseased Eye				Fellow Eye			
	Baseline (DB)	Final Visit (DF)	*p* (DB vs. DF)	Baseline (FB)	Final Visit (FF)	*p* (FB vs. FF)	*p* (DB vs. FB)	*p* (DF vs. FF)
NPA	19.16 ± 4.74	23.97 ± 2.60	0.0282 *	21.66 ± 3.21	23.13 ± 3.48	0.1109	0.1894	0.5676
FAZ	0.25 ± 0.10	0.32 ± 0.09	0.1146	0.39 ± 0.30	0.32 ± 0.12	0.5410	0.2418	0.8256
SCP_WHOLE	45.90 ± 3.34	45.33 ± 5.20	0.5593	48.30 ± 3.78	48.15 ± 5.06	0.9653	0.1850	0.3805
SCP_BRVO	43.83 ± 4.15	41.68 ± 7.73	0.3291	48.84 ± 3.54	47.46 ± 4.42	0.5866	0.0353 *	0.1244
SCP_UNAFFECTED	47.92 ± 3.44	47.32 ± 4.07	0.6601	48.76 ± 2.95	48.51 ± 5.71	0.8905	0.5782	0.6862
DCP_WHOLE	46.49 ± 1.47	46.60 ± 5.03	0.9870	48.17 ± 6.93	52.77 ± 4.98	0.1412	0.4699	0.0054 *
DCP_BRVO	42.92 ± 2.67	43.06 ± 5.88	0.9302	49.63 ± 8.17	51.51 ± 6.67	0.5449	0.0356 *	0.0024 *
DCP_UNAFFECTED	48.65 ± 1.50	48.36 ± 5.61	0.8859	48.23 ± 6.85	53.20 ± 4.14	0.1064	0.8932	0.0312 *

BRVO: Branch retinal vein occlusion, NPA: non-perfusion area, FAZ: foveal avascular zone, SCP: superficial capillary plexus, DCP: deep capillary plexus. * *p* < 0.05.

### 3.5. CCT Changes

In the aflibercept treatment group, mean CCT increased by 17.6 μm, whereas in the ranibizumab group it decreased by 8.75μm; however, these changes were not statistically significant (*p* = 0.28) due to the high variability within each group. There were no statistically significant differences in both groups between the diseased and fellow eyes (aflibercept group *p* = 0.07 and ranibizumab group *p* = 0.25).

### 3.6. Parameters Correlated to BCVA

SCP densities in both BRVO sectors and non-BRVO sectors were significantly correlated with BCVA in diseased eyes (Pearson’s Coefficient of Correlation (PCC) = −0.40, *p* < 0.05). NPA and DCP, in both BRVO sectors and non-BRVO sectors, were significantly correlated with BCVA for both diseased eyes (NPA, PCC = −0.24, *p* < 0.05; DCP, PCC = −0.49, *p* < 0.05) and fellow eyes (NPA, PCC = −0.51, *p* < 0.05; DCP, PCC = −0.55, *p* < 0.05) (Table 3).

In addition to univariate correlation, we also performed a multivariate analysis to further demonstrate the potential predictive role of these OCTA parameters on the visual outcome. The DCP density of the BRVO sector, in both the diseased and fellow eye, was found to be the predictive parameters influencing final BCVA in the final multivariate general linear model (GLM) analysis (Table 4).

## 4. Discussion

OCTA was approved by the Food and Drug Administration in 2016 to non-invasively produce images of the vascular layers of the retina, including the SCP and DCP [21]. It is useful for the description, quantification, and evaluation of treatments in retinal vascular diseases such as BRVO [13,14,15,16,17].

Several reports have demonstrated OCT findings as indicators of visual function and prognostic factors in BRVO-ME patients treated with anti-VEGF agents [22,23,24,25]. In the present study, final CRT and BCVA improved significantly after treatment. Regarding the diseased eye, NPA as well as SCP and DCP density of both BRVO and non-BRVO sectors were highly correlated with final BCVA. More importantly, the DCP density of the BRVO sectors also displayed strong prediction of final BCVA of the diseased eye. The improvements in anatomical and visual outcomes were rapid and sustained, with stable IOP through the follow-up period.

The SCP and DCP have been found to decrease in density in the BRVO sectors, as shown in previous studies, reflecting ischemic processes with retinal non-profusion in affected areas [13,14]. In our study, both SCP and DCP densities of BRVO sectors in diseased eyes remained persistently low after anti-VEGF treatment. These findings are consistent with previous studies on anti-VEGF therapy [14,15,16]. We hypothesized that although macular edema subsided and BCVA significantly improved, the ischemic damage in retinal capillaries had not recovered by the conclusion of the follow up period.

The DCP was much more affected than the SCP after anti-VEGF treatment, with statistically significant changes observed in comparison with the fellow eye. These differences may be due to the slight elevation of DCP in the fellow eye (Table 2). Aflibercept exerted more significant effects than ranibizumab. Previous studies have demonstrated that unilateral anti-VEGF injection may affect the contralateral eye due to systemic effects [26] and that aflibercept injections, but not ranibizumab, decrease systemic VEGF [27]. We speculated that the decreased VEGF levels in the fellow eye in the aflibercept group caused the redistribution of retinal capillary blood flow and subsequently elevated DCP. Other possible explanations include projection artifacts from other layers, and layer segmentation errors caused by shapes of the capillary networks and irregular boundaries [28]. We attempted to avoid these conditions by excluding patients with poor image quality. Further studies are warranted to investigate this condition.

Another factor contributing to this phenomenon is the structural difference between the SCP and DCP. The SCP has higher perfusion pressure due to direct connections with retinal arterioles, whereas the DCP is connected to macular superficial venules, which are more susceptible in BRVO [29]. In addition, the DCP is within the watershed-like area in which oxygen saturation is lower than in the inner or outer retina [30].

The effect of anti-VEGF therapy on the size of the NPA is still unclear. While some studies have found that anti-VEGF decreases the size of NPA and improves retinal flow [31,32], others have indicated otherwise [33,34]. In our study, the final NPA increased significantly. In detailed analysis, NPA decreased in the first and second months but subsequently increased, which suggested that retinal blood flow improved initially but that no collateral circulation was restored. Since VEGF is crucial for the establishment of collateral vessels, the blockade might result in a worse progression of retinal ischemia. High NPA was also correlated with higher risks of ME recurrence in a previous study [35].

Several studies have extensively discussed the correlation of BCVA and OCTA findings in RVO patients. Sellam et al. suggested that the density of the SCP and DCP in a diseased eye correlated with initial BCVA and final BCVA; whereas Balaratnasingam et al. demonstrated that the diameter of the FAZ was inversely correlated with BCVA [13,36]. In the present study, univariate correlation analysis found that SCP density in the diseased eye and NPA or DCP density in both eyes were significantly correlated with final BCVA. Multivariate model analysis confirmed that DCP density of BRVO sectors in both eyes was a strong predictive factor for visual outcome. Our findings suggest that DCP density in the fellow eye could serve as a new prognostic factor. We hypothesize that human eyes maintain symmetry and that the high capillary density of the fellow eye could be a sign that the diseased eye contains higher capillary restoration potential, which correlates with the final visual acuity of the diseased eye. Thus, OCTA enables the qualitative and quantitative evaluation of patients treated for BRVO.

Our study has a few limitations. First, our sample size was small due to the strict inclusion criteria. Second, the baseline BCVA was not equal between the ranibizumab and aflibercept groups, although this difference was not statistically significant. Third, the average number of injections were not the same between the ranibizumab and aflibercept groups, although this difference was also not statistically significant. For multivariate linear model analysis, we only included OCTA parameters into the model as predictors only, but not all of the available clinical data. This made the final model a balance between the inference model and the prediction model. This is a compromise between the current sample size and the obtaining of a practical working model. Finally, the lack of a true control group (untreated BRVO eyes) was also a crucial point. Considering the well-documented improvement in visual prognosis with anti-VEGF treatment, it was not realistic to include a group of subjects as an untreated control group. Further studies with larger sample sizes, longer follow-up periods, and more injections are needed to confirm our results. The investigation of new strategies to improve this vascular condition are also warranted.

## 5. Conclusions

OCTA enabled qualitative and quantitative evaluation bilaterally during the follow-up of our patients. In the diseased eye, NPA and SCP and DCP density of both BRVO and non-BRVO sectors were highly correlated with final BCVA. Moreover, in the fellow eye, DCP density of the BRVO sectors also displayed strong predictive power of final BCVA of the diseased eye. Densities of both SCP and DCP, especially in BRVO sectors, were persistently low compared to fellow eyes, even after treatment. Multimodal imaging, including FA, OCT, and OCTA, could provide the optimal approach for clinicians to monitor treatment-naïve patients with BRVO-ME.

## Figures and Tables

**Table 1:** The baseline demographics.

	All (*n* = 10)	Aflibercept (*n* = 5)	Ranibizumab (*n* = 5)	*p*
Age	61.40 ± 7.83	61.80 ± 10.26	61.00 ± 5.70	0.883
Gender (F)	5 (50%)	2 (40%)	3 (60%)	0.527
Eye (OD)	5 (50%)	3 (60%)	2 (40%)	0.527
SBP	151.60 ± 19.49	147.80 ± 20.90	155.40 ± 19.55	0.569
IOP	15.70 ± 2.91	14.20 ± 2.49	17.20 ± 2.68	0.104
CRT	396.60 ±135.64	367.60 ± 68.61	425.60 ±185.97	0.531
CCT	208.67 ± 94.02	214.20 ±129.33	201.75 ± 33.98	0.129
S	0.57 ± 1.48	0.67 ± 1.61	0.50 ± 1.62	0.932
C	−0.61 ± 0.61	−0.75 ± 0.90	−0.50 ± 0.41	0.418
BCVA	0.52 ± 0.30	0.67 ± 0.32	0.38 ± 0.22	0.832
NPA	19.16 ± 4.74	19.02 ± 5.71	19.30 ± 4.23	0.858
FAZ	0.25 ± 0.10	0.23 ± 0.08	0.28 ± 0.12	0.898
SCP	45.90 ± 3.34	45.88 ± 1.36	45.93 ± 4.82	0.637
DCP	46.49 ± 1.47	46.60 ± 1.38	46.38 ± 1.71	0.833
Total injections	5.80 ±2.53 (3.00~9.00)	7.00 ±2.12 (4.00~9.00)	4.60 ±2.51 (3.00~7.00)	0.141
Follow-up months	11.39 ± 2.14 (7.97~15.43)	11.72 ± 1.41 (9.53~13.30)	11.06 ± 2.84 (7.97~15.43)	0.654

F: female, OD: right eye, SBP: systolic blood pressure, IOP: intraocular pressure, CRT: central retinal thickness, CCT: central choroidal thickness, S: sphere, C: cylinder, BCVA: best-corrected visual acuity, NPA: non-perfusion area, FAZ: foveal avascular zone, SCP: superficial capillary plexus, DCP: deep capillary plexus.

**Table 3:** Correlations between OCTA parameters and BCVA (LogMAR).

		With LogMAR	
Side	Variable	PCC	*p*
**Diseased Eye**	NPA	−0.24546	0.0165 *
	FAZ	−0.04474	0.6668
	SCP (whole)	−0.40927	<0.0001 *
	SCP (BRVO)	−0.38861	0.0001 *
	SCP (unaffected)	−0.44414	<0.0001 *
	DCP (whole)	−0.49930	<0.0001 *
	DCP (BRVO)	−0.45791	<0.0001 *
	DCP (unaffected)	−0.42122	<0.0001 *
**Fellow Eye**	NPA	−0.51187	<0.0001 *
	FAZ	0.19560	0.0588
	SCP (whole)	−0.06948	0.5058
	SCP (BRVO)	−0.17612	0.0949
	SCP (unaffected)	−0.12261	0.2469
	DCP (whole)	−0.55549	<0.0001 *
	DCP (BRVO)	−0.58967	<0.0001 *
	DCP (unaffected)	−0.45140	<0.0001 *

Valm: visual acuity by LogMAR, Valm_diffr: relative change of Valm compared to initial exam during follow-up; PCC: Pearson’s Coefficient of Correlation, * *p* < 0.05. BRVO: Branch retinal vein occlusion, NPA: non-perfusion area, FAZ: foveal avascular zone, SCP: superficial capillary plexus, DCP: deep capillary plexus.

**Table 4:** Multivariate Analysis with Model Selection by General Linear Model (GLM).

GLM Model Selection Summary						
Effect	DF	Estimate	Adjusted R-Square	AIC	F Value	Pr > F
Intercept	1	1.37352	0	−151.4508	0	1
DCP of Affected Sector	1	−0.015374	0.152	−165.4669	17.13	<0.0001
DCP of Affected Sector, fellow eye	1	−0.009168	0.4375	−201.8591	46.19	<0.0001

Dependent variable of model: BCVA (LogMAR); Initial independent variables before final model obtained, including deep capillary plexus, affected and unaffected sectors; superficial capillary plexus, affected and unaffected sectors; fovea avascular zone, total retina avascular area, outer retina flow area; all of these parameters included data from both diseased and fellow eyes. In addition to the original data obtained during every clinical visit, a change in data compared to the initial data was also included in the parameter list of model analysis. BCVA, best corrected visual acuity; DF, degree of freedom; AIC, Akaike information criterion; Pr, probability; DCP, deep capillary plexus.

## Data Availability

Not applicable.
